# Generation of hepatoma cell lines deficient in microsomal triglyceride transfer protein

**DOI:** 10.1016/j.jlr.2022.100257

**Published:** 2022-08-02

**Authors:** Narasimha Anaganti, Atrayee Chattopadhyay, John T. Poirier, M. Mahmood Hussain

**Affiliations:** 1 Department of Foundations of Medicine, NYU Long Island School of Medicine, Mineola, NY, USA; 2 Perlmutter Cancer Center, New York University Langone Health, New York, NY, USA; 3 VA New York Harbor Healthcare System, Brooklyn, NY, USA

**Keywords:** electroporation, ribonucleoprotein complex, gene ablation, apoB100, lipoprotein assembly and secretion, CRISPR/Cas9, Huh-7 cells, transfection, missense mutations, lipid transfer, ApoB, apolipoprotein B, CRISPR/Cas9, RNA-guided clustered regularly interspaced short palindromic repeats associated sequence 9, gRNA, guide ribonucleic acid, indels, insertions and deletions, ms, millisecond, MTP, microsomal triglyceride transfer protein (gene, *MTTP*), PDI, protein disulfide isomerase, RFP, red fluorescent protein, RNP, ribonucleoprotein, sgRNA, single guide ribonucleic acid, TAG, triacylglycerol

## Abstract

The microsomal triglyceride transfer protein (MTP) is essential for the secretion of apolipoprotein B (apoB)48- and apoB100-containing lipoproteins in the intestine and liver, respectively. Loss of function mutations in MTP cause abetalipoproteinemia. Heterologous cells are used to evaluate the function of MTP in apoB secretion to avoid background MTP activity in liver and intestine-derived cells. However, these systems are not suitable to study the role of MTP in the secretion of apoB100-containing lipoproteins, as expression of a large apoB100 peptide using plasmids is difficult. Here, we report a new cell culture model amenable for studying the role of different MTP mutations on apoB100 secretion. The endogenous *MTTP* gene was ablated in human hepatoma Huh-7 cells using single guide RNA and RNA-guided clustered regularly interspaced short palindromic repeats-associated sequence 9 ribonucleoprotein complexes. We successfully established three different clones that did not express any detectable *MTTP* mRNA or MTP protein or activity. These cells were defective in secreting apoB-containing lipoproteins and accumulated lipids. Furthermore, we show that transfection of these cells with plasmids expressing human *MTTP* cDNA resulted in the expression of MTP protein, restoration of triglyceride transfer activity, and secretion of apoB100. Thus, these new cells can be valuable tools for studying structure-function of MTP, roles of different missense mutations in various lipid transfer activities of MTP, and their ability to support apoB100 secretion, compensatory changes associated with loss of MTP, and in the identification of novel proteins that may require MTP for their synthesis and secretion.

Microsomal triglyceride transfer protein (MTP) is a heterodimer of 97 kDa subunit MTP and 55 kDa subunit protein disulfide isomerase (PDI). MTP plays a major role in assembly and secretion of apolipoprotein B (apoB)-containing lipoproteins, chylomicrons, and VLDL in the intestine and liver, respectively (1, 2). Loss of function mutations in the *MTTP* gene results in abetalipoproteinemia (3). Since the discovery of MTP as an essential protein in the secretion of apoB-containing lipoproteins, heterologous cell expression systems have been used to establish the role of MTP in apoB secretion (4). Our group has been studying evolution (5, 6, 7) and structure-function (8, 9, 10) of MTP to understand the molecular basis for the loss of function missense mutations in abetalipoproteinemia patients. In these studies, we transfected Cos-7, monkey kidney, cells with plasmids expressing wild type and mutant MTP. These studies showed that all abetalipoproteinemia missense mutations lacked both triglyceride and phospholipid transfer activities (8, 9, 10). PDI binding studies identified mutations that were defective in PDI binding and lacked lipid transfer activities indicating that association of the MTP subunit with PDI is critical for lipid transfer activities. These studies also identified missense mutations that interacted well with PDI and yet were deficient in lipid transfer activities indicating that different residues, and possibly domains, in MTP are also required for lipid transfer activity. Besides these studies, we also examined the capacity of different MTP missense mutations in supporting apoB secretion. These studies revealed that deficiency of lipid transfer activities was always associated with no apoB48 secretion (8, 9, 10). A major drawback of these studies was that we were unable to study the effect of different mutant MTPs on the secretion of apoB100 (8, 9, 10). ApoB100 is a large protein of about 4,560 amino acids, and the mRNA is about 15 kb. Thus, expression of apoB100 using plasmids is difficult (11). Therefore, we made apoB100 chimeric proteins with different fluorescent tags to enhance detection (12). However, these methods also had very little success, as transfection of large plasmids expressing apoB100 was difficult. In this study, we tried a different approach. We hypothesized that ablation of the *MTTP* gene in human hepatoma cells might avoid the need to transfect large plasmids expressing human apoB100.

There are various methods to ablate genes in cells. Homologous recombination is the traditional method for gene knockout in which an antibiotic resistance marker replaces the target gene. In this procedure, the transfected plasmid carries homologous flanking sequence of target gene to replace endogenous sequence with a marker gene *via* homologous recombination. However, homologous recombination is an inefficient process and accounts for only 10^−2^ to 10^−3^ of DNA integrations in diploid and polyploid cell lines; hence, multiple cycles of transfections are required to delete all alleles (13, 14). In other methods, site-specific nucleases that cleave the target gene and make double strand breaks are used. Zinc finger nucleases (13, 14) and transcription activator-like endonucleases are the two enzymes widely used for gene knockouts (15, 16). Recently, RNA-guided clustered regularly interspaced short palindromic repeats (CRISPR)-associated sequence 9 (CRISPR/Cas9) system has become a powerful tool for targeted gene disruption and editing (17). CRISPR/Cas9 process consists of identifying target-specifying guide RNA (gRNA) and expressing them with Cas-9 enzyme, an RNA-guided endonuclease that cleaves both strands of target DNA. After cleavage in both DNA strands, the nonhomologous end joining DNA repair mechanism stitches back the cleaved DNA. This process introduces insertions/deletions (indels) at the cleavage sites (18, 19, 20, 21) causing frame shift mutations that create early stop codon and premature termination of protein translation (17, 22, 23). Strategies have been developed using gRNA with CRISPR-Cas9 technology that minimizes off-target cleavage (15, 24). Due to simplicity in designing gRNAs, high specificity and precision, minimization of off-target cleavage, and ability to accommodate a variety of cell types and organisms, CRISPR/Cas9 is a preferred method for gene editing.

Different CRISPR-based platforms are available for generating knockout cells. All-in-one plasmid-based systems express gRNA and Cas9 nuclease using a single plasmid (17). Low plasmid-based transfection efficiency is a major limitation of this method. To overcome this limitation, Cas9 protein preassembled with gRNA as ribonucleoprotein (RNP) complexes have been developed for achieving efficient gene editing (23) and for genome wide screening studies (25). RNP complex–mediated editing enhances the rate of gene insertion. Furthermore, the shorter half-life of the Cas9 RNP leads to fewer off-target events (23).

We used both plasmids and RNP-based approaches to ablate MTP in Huh-7 cells. We report generation of three MTP knockout cells (MKOs) using RNP complexes of CRISPR/Cas9 and specific single guide RNAs (sgRNAs). These MKO cells do not express detectable levels of MTP mRNA, protein and activity; moreover, they are defective in apoB100 secretion. Re-expression of human MTP partially restored MTP mRNA, protein, and activity levels as well as apoB100 secretion. These MKO cells can be valuable tools in screening different MTP mutants for their ability to support apoB100 secretion.

## Materials and methods

### Materials

Human hepatoma Huh-7 cells and plasmids pcDNA3 and pcDNA3-hMTP-FLAG have been described previously (5, 9, 10, 26, 27). The MTP CRISPR/Cas9 KO plasmid containing MTP gRNAs (#SC-401884) and MTP-HDR plasmid (#SC-401884-HDR, Santa-Cruz Biotechnology) that contains sites for homologous recombination with the endogenous *MTTP* gene and genes encoding for red fluorescent protein (RFP) and puromycin resistance. SpCas9 2NLS Nuclease and Custom sgRNAs were from Synthego. Oligonucleotides were obtained from Integrated DNA Technology. Reverse transcriptase (Applied Biosystems, # A46109) and qPCR kits were obtained from Eurogentec (# RT-SN10-05). Anti-FLAG® M2 affinity agarose gel (#A2220-5ML) and other chemicals were purchased from Millipore Sigma (St. Louis, MO). Polyclonal anti-hMTP antibody was from Abcam (# ab63467). FLAG peptide was custom synthesized (GenScript). Round bottom black 96-well assay plates were obtained from Costar (#3792, Kennebunk, ME). Mouse LDL (Apo-B) monoclonal antibody (Clone 1D1) was purchased from Mybiosource (# MBS465020). Goat polyclonal anti-hApoB antibody was from Academy Bio-medical Company Inc. (#20S-G2-1.5ml). Swine anti goat-IgG antibody was from Southern Biotech (#6300-04). qPCR plates were from Applied Biosystems (#4346906), and 96-well ELISA assay plates were bought from Corning (#3366). Puromycin was purchased from Gibco (# A1113803).

### Transfection of Huh7 cells by chemical reagent

To knockout the *MTTP* gene by plasmid-based CRISPR system, plasmids expressing gRNA and CRISPR/Cas9 KO plasmids were co-transfected with MTP-HDR plasmids (1:1 ratio) into Huh-7 cells by chemical method using EndoFectin Max transfection reagent (Genecopoeia, #EF013) as described earlier (8, 10, 27). Huh-7 cells were seeded in T75 flasks and allowed to grow until they reached 80%–90% confluence. Cells were trypsinized, washed with OptiMem media, and 10^6^ cells were resuspended in 5 ml of OptiMem media. The MTP CRISPR/Cas9 KO (5 μg), MTP-HDR plasmids (5 μg), and EndoFectin (20 μl) were mixed in 0.5 ml of OptiMem media. The mixture was incubated at room temperature for 10 min and added to 90 mm cell culture dishes. Cells were then added to the plate. After 12 h incubation in CO_2_ incubator at 37°C, the OptiMem media were removed, and DMEM media was added. Similarly, the transfection of pcDNA3 or pcDNA3-hMTP-FLAG (9 μg of each) into Huh7 MTP KO cells was carried out using EndoFectin Max transfection reagent (Genecopoeia, #EF013).

### Screening of colonies for puromycin resistance and RFP expression

To select cells with gene deletions after plasmid based method, we first determined the amounts of puromycin needed to kill cells. Huh-7 cells were titrated for puromycin resistance by growing them (10^4^ cell per well) in a 96-well microtiter plate in the presence of 0–10 μg/ml of puromycin. At 2 μg/ml and higher puromycin concentrations, no growth was observed. To obtain gene knockout cells, Huh-7 cells were transfected with MTP CRISPR/Cas9 KO and MTP-HDR (contains puromycin resistance and *rfp* genes) plasmids and initially grown in media without antibiotic. After 48 h, media were removed, and fresh media with 1 μg/ml puromycin were added and allowed to grow for another 48 h. Subsequently, puromycin concentration was increased to 2 μg/ml. This was repeated three times to remove untransfected cells. The remaining puromycin resistance cells were trypsinized, serially diluted, and seeded in 96-well microtiter plate (100 μl/well) so that each well gets one cell. The plate was incubated in the presence of 2 μg/ml of puromycin for 3–4 weeks to allow colony growth. The colonies were visualized using a fluorescent microscope for RFP and used to measure MTP activity and protein levels.

### gRNA design, RNP complex formation, and transfection by electroporation

We targeted 200 bp within exon 2 of the *MTTP* gene for gene ablation. Three gRNAs were designed using CRISPR design tool (Synthego). Based on off target score, top three sequences gRNA1-CUUUAUGCAGGUCACACAAC, gRNA2-CCACUGAAGUUCUUCUUGAU, and gRNA3-CAUCCACGUUGGAGGAAAUG were selected. Next, three synthetic sgRNAs were synthesized (Synthego). Each sgRNA consisted of gRNA sequence along with Cas9 nuclease recruiting sequence that facilitates binding of the RNA to Cas9 nuclease. To prepare RNP, we incubated SpCas9 2NLS Nuclease enzyme (2.0 μmol) with three sgRNA (2.5 μmol) in 5 μl PBS in a PCR tube and incubated for 15 min at room temperature. For transfection, Huh-7 cells were grown to 80%–90% confluence, and media were changed 16–18 h before the trypsin treatment of the cells. Trypsinized cells were washed twice with PBS by centrifugation at 5,000 rpm (∼1,000 *g*) for 5 min at 4°C. The RNP complex (5 μl) was mixed with 10^5^ Huh-7 cells suspended in 10 μl of buffer R (Neon transfection system 10 μl kit, # MPK1096, ThermoFisher Scientific) to get a final volume of 15 μl. The RNP complex was transfected in Huh-7 cells by electroporation. For electroporation, 10 μl of Huh-7 cells and RNP complex mixture were aspirated into gold plated tips using pipette supplied with kit (# MPK1096, ThermoFisher Scientific). The tip containing cells and RNP mixture was placed in electroporation cells and exposed to different electric pulses of 0 V, 800 V, 1,250 V, 1,450 V, and 1,650 V for 10 milliseconds (ms) using Neon transfection system (ThermoFisher Scientific, #MPK5000) as per the manufacturer protocol. Then, cells were immediately transferred to prewarmed media in 6-well plates and incubated in CO_2_ incubator at 37°C. Cells were then grown without antibiotic until they reach 80%–90% confluence for about 5–7 days. The media were changed every day.

### PCR amplification of target exon and its DNA sequencing

Transfected Huh-7 cells were grown to 80%–90% confluence. These cells were trypsinized and divided into three parts. One part was serially diluted and seeded in 96-well plate to obtain single cell per well, second part was used to isolate chromosomal DNA, and third part was frozen for storage. The gRNA target site in exon 2 was PCR amplified using upstream forward primer (F1 5′-GTGAGACAGCATGTTCCCTTAC-3′) and downstream reverse primer (R1 5′-TGCATTTATCTGGTAGAAAATGC-3′). The PCR products were resolved on 2% agarose gel. The same PCR products of all transfected cells including untransfected controls were sequenced using F1 primer (Genewiz, NJ). The chromatogram was analyzed using SnapGene and Chromas softwares. To identify mutations in selected clones, exon 2 was amplified using these primers, sequenced, and aligned using SnapGene.

### ApoB measurement in the media by ELISA

Previously described ELISA methods estimated apoB100 concentrations in the media (10, 28). In brief, the media were collected from cells grown either in 24-well, 6-well, or 100-mm cell culture plates. Media were centrifuged at 12,000 rpm (13,500 *g*) for 5 min to remove dead cells and particulate debris, diluted appropriately, and stored at −80°C freezer until use. The ELISA plates were coated with 100 μl of capture antibody (1:10,000 dilution of mouse monoclonal anti-ApoB antibody 1D1, 1 mg/ml) in PBS and incubated overnight at 4°C. Next morning, plates were washed thrice with 350 μl of PBST buffer and blocked with 100 μl of 3% BSA solution (in PBS buffer) for 1 h at room temperature. Then plates were washed as above and 100 μl of diluted and/or undiluted samples were loaded along with 0–100 ng of LDL standard and incubated at 37°C for 2 h. The plates were washed thrice with PBST, and then 100 μl of detection antibody (goat polyclonal anti-ApoB antibody, 1:1,000 dilution) was added to each well and incubated for another 1 h at 37°C. Again, plates were washed thrice, and 100 μl of secondary antibody alkaline phosphatase-labeled swine polyclonal anti-goat IgG antibody (1:2,000 dilution, #6300-04, Southern Biotech) was added to each well and further incubated for 1 h at 37°C. Finally, the plate was washed, and color was developed by adding 100 μl of 1 mg/ml (w/v) of p-nitrophenyl phosphate substrate dissolved in diethylamine buffer (100 mM glycine, 1 mM MgCl_2_.6H_2_O, and 1 mM ZnCl_2_, pH 10.4) and incubating in the dark for 5–10 min or until the yellow color developed. The yellow color was measured at 405 nm. The concentrations of apoB in samples were calculated using the LDL standard curve generated in parallel in the same plate.

### RNA isolation and RT-qPCR

The total RNA from control and MTP KO Huh-7 cells was isolated using TRIzol reagent (ThermoFisher Scientific, # 15596018) according to manufacturer’s protocol. The RNA quality and quantity was assessed by Nanodrop (ThermoFisher Scientific, # ND-ONE-W). The cDNA was synthesized using random primers and 2 μg of total RNA using SuperScript IV CellsDirect cDNA Synthesis Kit (Invitrogen, # 11750150) as per manufacturer’s protocol. For RNA controls, the same amount of RNA was used without reverse transcriptase. Expressions of MTP and reference gene 18S in control and MTP-KO cells were analyzed by quantitative real time PCR (qPCR). The qPCR was performed using appropriately diluted cDNA and RNA controls using 2× qPCR kit (Eurogentec, #RT-SN10-05) in Real time PCR machine QuantStudio3 (Applied Biosystems, #A28131).

### MTP expression, purification, activity assay, and binding to PDI

The plasmid-based MTP expression in Huh-7 MTP KO cells was done as follows. Cells were grown in 6-well plates until 90% confluent. pcDNA3 or pcDNA3 carrying hMTP cDNA (3 μg) were transfected using EndoFectin Max transfection reagent (Genecopoeia, #EF013). The expression of hMTP protein in wild type Huh-7 cells as well as MTP KO cells transfected with pcDNA3 carrying MTP cDNA were analyzed by MTP activity and Western blot techniques as described earlier (10, 27). In brief, cells were lysed in buffer K by sonication with pulse of 2 s on 1 s off for 90 s and centrifuged at 12,000 rpm (13,500 *g*) at 4°C for 10 min to remove unbroken cells and cell debris. The clear lysates (50 μg) were used for MTP triglyceride transfer assay as before (8, 27, 29, 30). In addition, approximately 300 μg of crude protein was used for MTP purification using anti-Flag M2 affinity gel (Sigma, A2220-5ML). The bound protein was eluted using 100 μM of flag peptide (custom synthesized from Genscript) as described earlier (10, 27).The crude protein (25 μg) and purified MTP protein (25 μl) were resolved on 8% SDS-PAGE and blotted on to nitrocellulose membrane. The MTP and PDI monomer subunits were detected using rabbit polyclonal anti-hMTP antibodies (Abcam, #ab63467) and mouse monoclonal anti-PDI antibodies (Invitrogen, #RL77), respectively. Actin, the loading control, was detected using rabbit polyclonal anti-β-actin antibodies (Cell signaling, #4967S).

### Cell proliferation assay

The cell proliferation of MTP-KO cells was monitored using Cell Titer 96 Aqueos One Solution (Promega, # G3580) as per manufacturer’s protocol. In brief, 10,000 cells were seeded in each well of 96-well microtiter plate. The cell viability was assessed at 24 h and 48 h. For this purpose, media were removed from wells, and 300 μl of media containing assay reagent were added and incubated for 3 h at 37°C. The purple color of formazan crystals formed was measured colorimetrically at 490 nm.

### Measuring lipid accumulation in MKO cells

For studying lipid accumulation due to MTP knockout, MKO-3 cells were cultured for 96 h and the following studies were performed.

#### Lipid droplet and ER staining

Cells were plated on chambered glass slides (Falcon, Corning) and incubated in 500 μl growth medium per chamber. Immediately prior to staining, the media were removed from each chamber, cells were washed with PBS (pH 7.4), and then fixed with 3% (*v/v*) formaldehyde. For performing immunofluorescence, the fixed cells were washed with PBS, permeabilized with 0.3% (v/v) Triton-X, and then incubated with 5% goat serum for 1 h at room temperature. Subsequently anti-calnexin antibody (1:250, Abcam) was added to the cells. After 2 h incubation, cells were rinsed with PBS, and treated with goat anti-rabbit IgG, Alexa Fluor 633 (1:500, Life Technologies) for 1 h. For staining the lipid droplets, cells were rinsed again with PBS and incubated with 10 μg/ml BODIPY® 493/503 in dark. The stained cells were then washed with PBS, and the chamber was slowly removed before mounting the cover slips using ProLong® Gold antifade reagent with DAPI (ThermoFisher Scientific). The slides were then imaged under Nikon Eclipse Ti confocal microscope with 400× magnification.

#### Oil Red O staining

Huh-7 and MKO-3 cells were cultured for 96 h in 24-well plates, washed three times with PBS, and incubated with Oil red O stain (0.36% in 60% isopropanol) for 20 min. Cells were rinsed with PBS to remove the excess stain and imaged using Nikon Eclipse TE300 microscope under 40× magnification. For quantification, the Oil red O from the stained cells was extracted in 100% isopropanol, and absorbance was measured at 490 nm.

#### Triglyceride extraction and quantification

Cells (10^5^) were plated in 6-well plates and incubated in growth medium for 96 h. The total triacylglycerol (TAG) was extracted by incubating the cells in 1 ml of 100% isopropanol overnight at 4°C. The isopropanol from each well was subsequently collected in microcentrifuge tubes, dried, and the TAG was resuspended in 100 μl isopropanol. For quantification, 10 μl from each sample was added to a 96-well plate followed by the addition of 90 μl of triglyceride reagent (Pointe Scientific). Absorbance was measured at 490 nm.

### Bioinformatics and statistical analysis

Specific gRNAs were designed using CRISPR design tool (Synthego). Specific primers were designed using SnapGene software. The Sanger sequence results were analyzed using SnapGene and Chromas software. The levels of MTP and actin transcripts were calculated using 2^−ΔΔCt^ method. The percentage transfer (%T) of TAG was calculated as previously described (29, 30) using the formula [%T = (Fs − Fb)/(Ft − Fb) × 100], where Fs is the fluorescence of the test sample, Fb is the fluorescence of the blank, and Ft is total fluorescence. All graphing and statistical analyses were performed in GraphPad Prism software, versions 8 and 9. All data are presented as mean ± SD. The symbols ∗, ∗∗, ∗∗∗, and ∗∗∗∗ represent significance at *P* < 0.05, *P* < 0.01, *P* < 0.001, and *P* < 0.0001, respectively.

## Results

### Plasmid based expression of sgRNAs and Cas9 in hepatoma cells to generate MKO cells

**Fig. 1:**
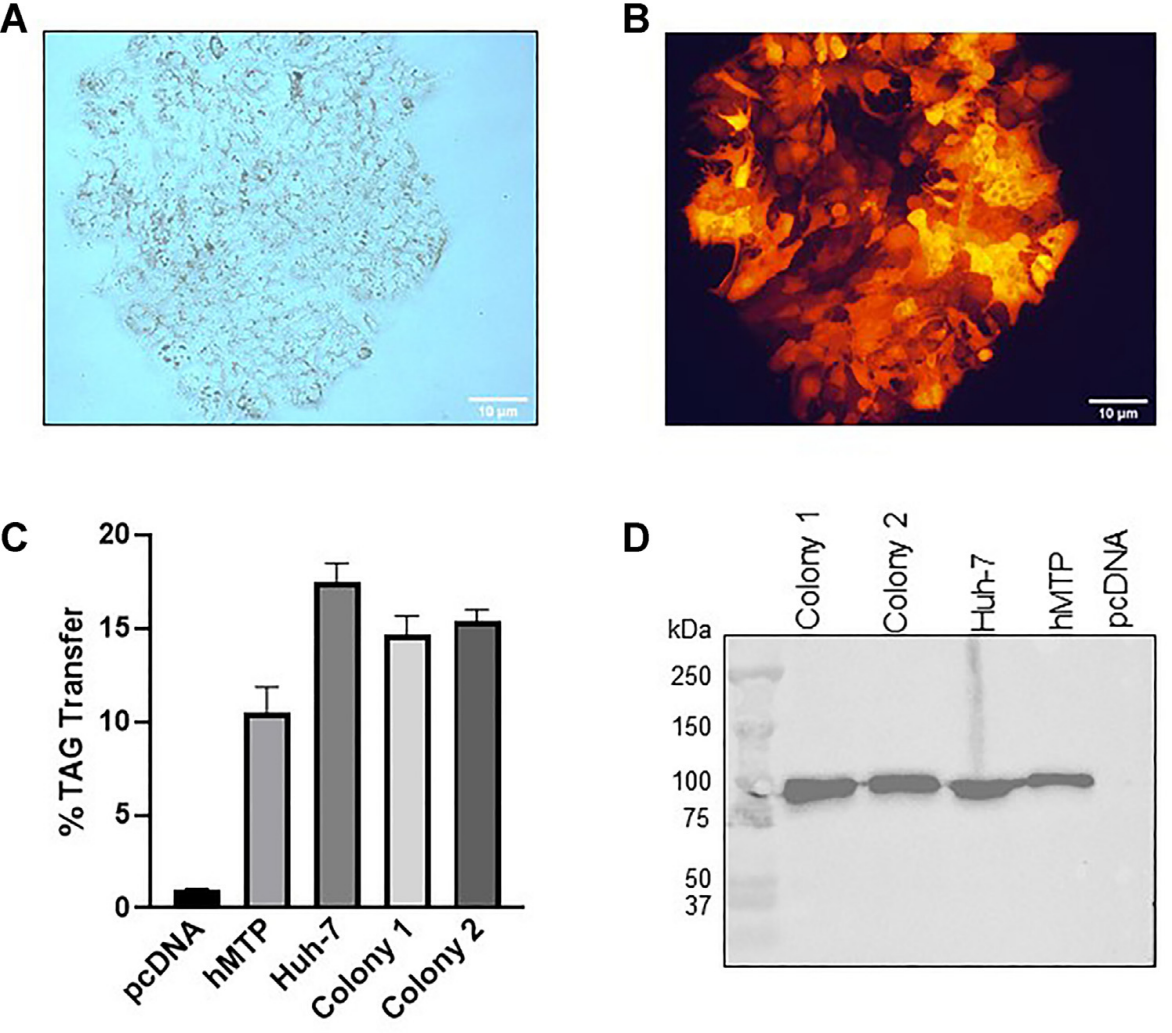
Gene knockout using plasmid based CRISPR/Cas9 expression. Huh-7 cells were cotransfected with plasmids carrying three gRNAs and Cas9 gene with MTP-HDR plasmid containing flanking sequences with Cas9 cutting sites to replace the *MTTP* gene by puromycin resistance and *rfp* genes and were cultured in the presence of puromycin (2 μg/ml). As expected, few puromycin-resistant and RFP expressing colonies were obtained. A: Light microscope image showing a colony of cells. B: Fluorescence microscope image shows presence of RFP (10× objective lens). C: The triacylglycerol (TAG) transfer activity of MTP in the cell lysates of two puromycin resistant, RFP positive colonies along with wild type Huh-7 cells. pcDNA and hMTP refer to Cos-7 cells transfected with pcDNA3 control plasmid and human MTP. D: The MTP protein levels in these cells were detected by anti-hMTP antibodies. MTP protein and activity was normal in puromycin-resistant colonies indicating no knockdown. CRISPR/Cas9, RNA-guided clustered regularly interspaced short palindromic repeats–associated sequence 9; gRNA, guide ribonucleic acid; MTP, microsomal triglyceride transfer protein (gene, MTTP); PDI, protein disulfide isomerase; RFP, red fluorescent protein.

Human hepatoma Huh-7 cells express apoB and MTP and secrete apoB-containing lipoproteins similar to HepG2 cells (31). We have recently used these cells to identify microRNA-30c analogs that reduce MTP expression (32). In our experience, these cells are easier to transfect than HepG2 cells. Therefore, we selected these cells for MTP ablation. First, we attempted to make MTP deficient Huh-7 cells using plasmid-based expression of 3 gRNAs and CRISPR/Cas9 with co-transfection of an MTP-HDR plasmid containing flanking sequences of Cas9 cutting sites, puromycin antibiotic resistance, and RFP genes. We obtained few colonies with puromycin resistance and RFP (Fig. 1A, B) indicating that homologous recombination did occur. However, these cells had normal MTP activity and protein (Fig. 1C, D) levels indicating that these colonies were not MTP deficient.

### gRNA design and RNP complex to delete MTP in human hepatoma Huh-7 cells

**Fig. 2:**
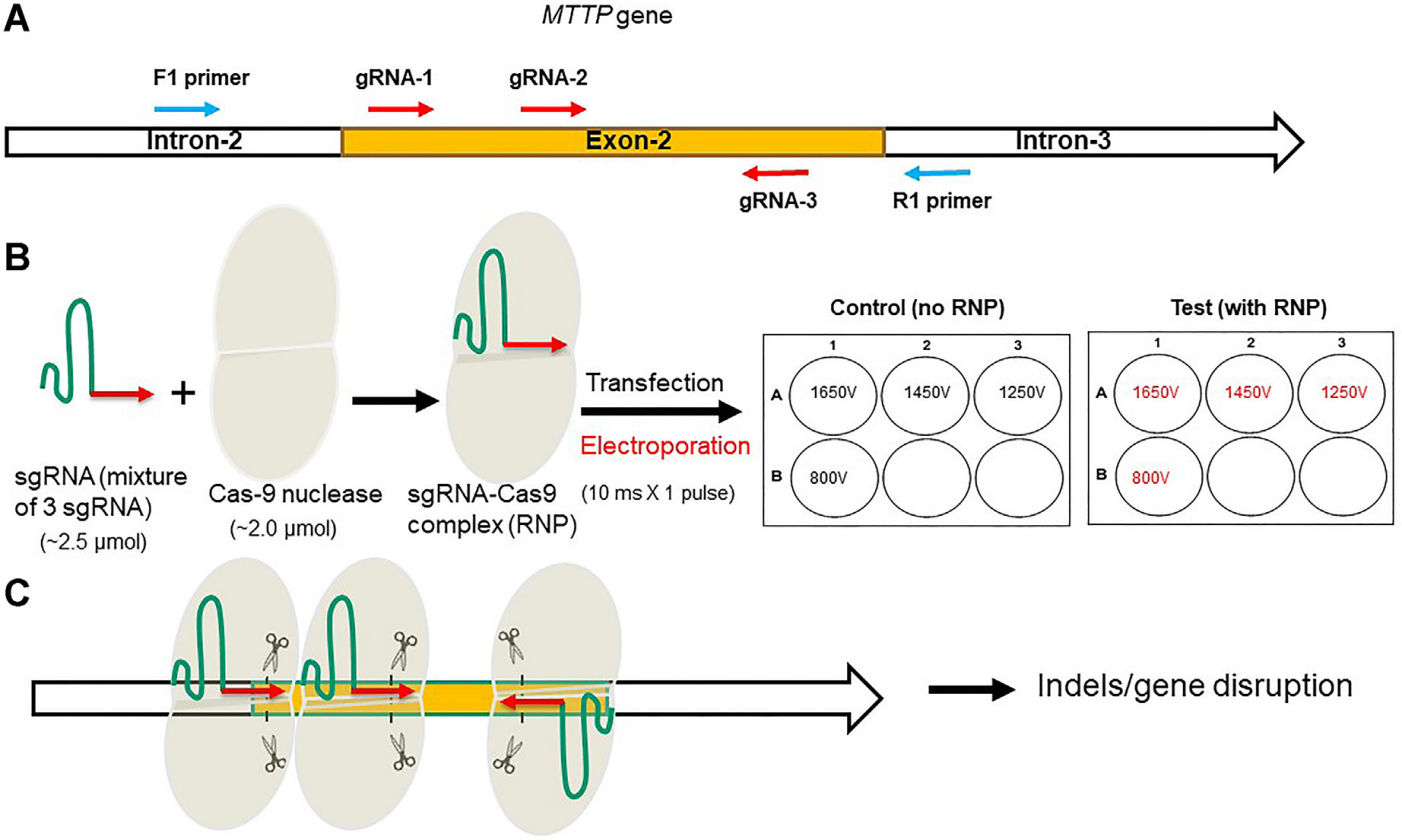
Guide RNA design, RNA–protein complex formation and electroporation. A: A schematic representation of exon 2 of human *MTTP* gene. Three different guide RNAs (gRNAs) (red arrows) were designed within exon 2 for targeting. Forward and reverse primers (blue arrows) were used for amplification of the exon 2. B: Single guide RNA (sgRNA) consists of gRNA (red) and a recruiting sequence (green) for Cas9 enzyme. The RNP of sgRNA and Cas-9 protein complex was made in vitro by mixing 2.5 μmol of sgRNA and 2.0 μmol of Cas-9 protein in a microcentrifuge tube. This mixture was transfected into Huh-7 cells by electroporation using various electric pulses of different voltages (800 V, 1,250 V, 1,450 V, and 1,650 V) for 10 ms each. The control cells were also given similar pulses without RNP. The electroporated cells were resuspended in prewarmed media and plated in 6-well plate. C: A schematic representation showing the binding of gRNAs and expected cleavage sites on the target DNA. Cleavage followed by repair is expected to cause indels at cleavage site leading to gene disruption. CRISPR/Cas9, RNA-guided clustered regularly interspaced short palindromic repeats–associated sequence 9; RNP, ribonucleoprotein; sgRNA, single guide ribonucleic acid.

Next, we used Cas9 protein and sgRNA complex to achieve *MTTP* gene ablation. We identified three regions within 200 bp of the *MTTP* exon 2 for the synthesis of gRNA (Fig. 2A) and synthesized three sgRNAs containing the targeting gRNA sequence along with Cas9 nuclease-recruiting sequence (Fig. 2B). These sgRNA and Cas9 proteins were mixed to make the RNP complexes (Fig. 2B) and introduced these complexes by electroporation into Huh-7 cells. For electroporation, single electric pulse of 800 V, 1,250 V, 1,450 V, or 1,650 V was given for 10 milliseconds (Fig. 2B) to cells in the presence of RNP complexes. After pulse, cells were immediately transferred to prewarmed media in 6-well plates (Fig. 2B) and incubated until the plate became 90% confluent. Once the RNP complex enters the nucleus, gRNA binds to homologous sequences in the target gene, and Cas9 cuts the DNA. Ligation of the DNA causes indels as shown in schematic diagram (Fig. 2C).

### Primary screening for the *MTTP* gene disruption

**Fig. 3:**
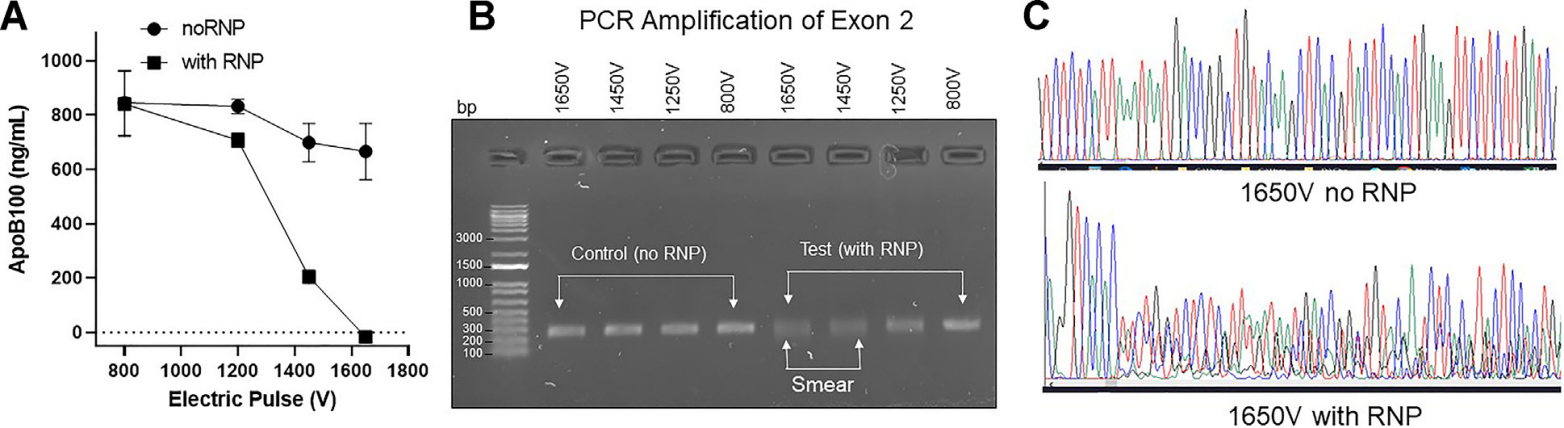
Primary screening for indels. A: The RNP and no RNP transfected cells pulsed with different voltages were grown for 7 days with media changing every day until cells became 90% confluent. On the seventh day, media were collected to measure apoB100 levels using ELISA. B: Genomic DNA was isolated (genomic DNA isolation kit, NEB) from cells. The gRNA target sequence of about 250 bp was PCR amplified using forward and reverse primers (blue arrows) shown in Fig. 2A. These amplicons were resolved on 2% agarose gel. First four lanes (left) represent controls, and second four lanes (right) represent the test samples (RNP-transfected cells). The diffuse DNA bands in two lanes (1,650 V and 1,450 V) identified with white line represent indels. C: A representative chromatogram of DNA sequence from cells transfected using 1,650 V with no RNP (top) and with RNP (bottom). apoB100, apolipoprotein B100; gRNA, guide ribonucleic acid; RNP, ribonucleoprotein.

MTP is essential for the secretion of apoB-containing lipoproteins (1, 2). Thus, absence of apoB in the media would be an indication for the loss of MTP. Therefore, we measured apoB in media (Fig. 3A). There was no difference in apoB secretion between control and either RNP or no RNP-transfected cells pulsed with 800 V (Fig. 3A). With 1,250 V pulse, we observed only 10%–12% reduction in apoB secretion in RNP-transfected cells compared to nontransfected cells. About 75% loss in apoB secretion occurred when 1,450 V pulse was used during electroporation. The complete loss of apoB secretion was observed at 1,650 V pulse. When cells were pulsed with 1,450 V and 1,650 V in the absence of RNP, about 5%–10% reduction in apoB secretion was seen compared with the untransfected control cells. These results indicate that reduction in apoB secretion was the highest at 1,650 V, and no or very low transfection occurred at 800 V and 1,250 V. These studies show that as the pulse intensity increases, the transfection and MTP gene ablation efficiency increases as measured by decreases in apoB secretion (Fig. 3A).

To confirm ablation of the *MTTP* gene, genomic DNA was isolated from these polyclonal cells, and the target sequence in exon 2 was PCR amplified (Fig. 3B). Tight bands were seen in controls, 800 V, and 1,250 V pulsed cells (Fig. 3B) indicating for the absence of indels. In contrast, there were smears of DNA bands in cells transfected with RNP using 1,450 V and 1,650 V. Sequencing of PCR products from these cells revealed overlapping chromatogram (Fig. 3C) strongly suggesting for changes in the target DNA sequence most likely due to indels.

### Selection of individual knockout colonies based on apoB100 secretion

**Fig. 4:**
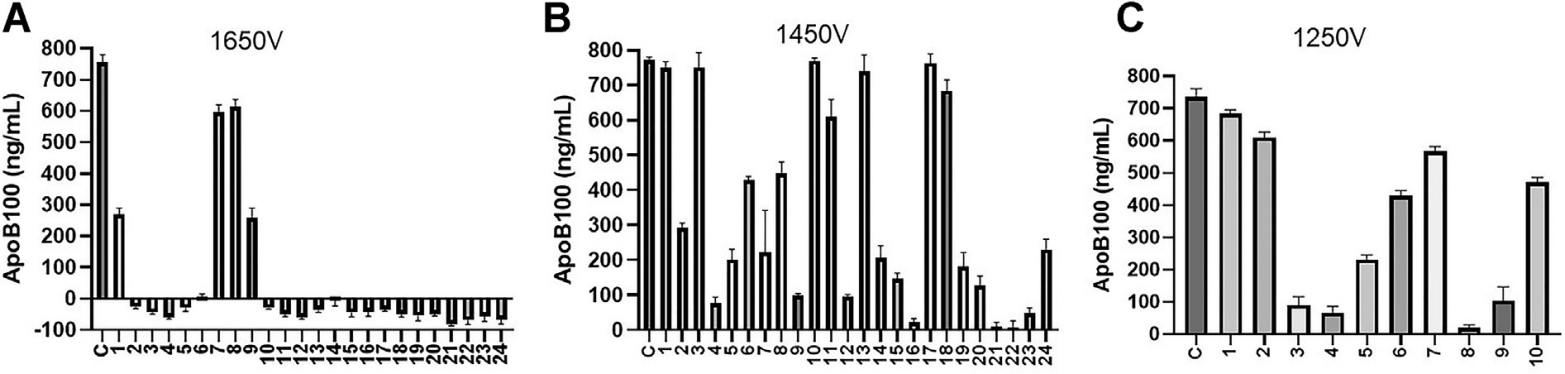
Screening of individual colonies based on apoB100 secretion. The RNP transfected plates using 1,250 V, 1,450 V, and 1,650 V were trypsinized, diluted, and seeded in a 96-well plate in such way that each well gets one cell. These plates were incubated for 3–4 weeks until the media color changed to orange/yellow indicating colony growth. In addition, colony growth was visualized under microscope. Well-grown colonies were transferred to individual wells in a 24-well plate and allowed to grow for 1 week with media changes every 48 h until the wells become > 90% confluent. The secreted apoB100 was measured in triplicate. ApoB100 concentration in the media of cells electroporated using (A) 1,650 V, (B) 1,450 V, and (C) 1,250 V pulses. “C” represents control cells that were electroporated without RNP. Numbers refer to different selected colonies. apoB100, apolipoprotein B100; RNP, ribonucleoprotein.

Next, cells were plated at high dilutions to obtain approximately one cell per well in 96-well plates. We were able to amplify 32, 30, and 20 colonies from 1,650 V, 1,450 V, and 1,250 V plates. These colonies were transferred to 24-well plates for further amplification of cell numbers. After a week, media from 24 colonies each of 1,650 V and 1,450 V and 10 colonies of 1,250 V plate were collected, and apoB concentrations were measured by ELISA. Twenty colonies from 1,650 V transfected cells showed no apoB secretion (Fig. 4A). In 1,450 V plate, seven colonies lost ≥90% apoB secretion (Fig. 4B). In 1,250 V plate, only four colonies showed >90% reduction in apoB secretion (Fig. 4C). These results indicated for the presence of several colonies that might be deficient in MTP function.

### Determination of MTP protein expression and its activity

**Fig. 5:**
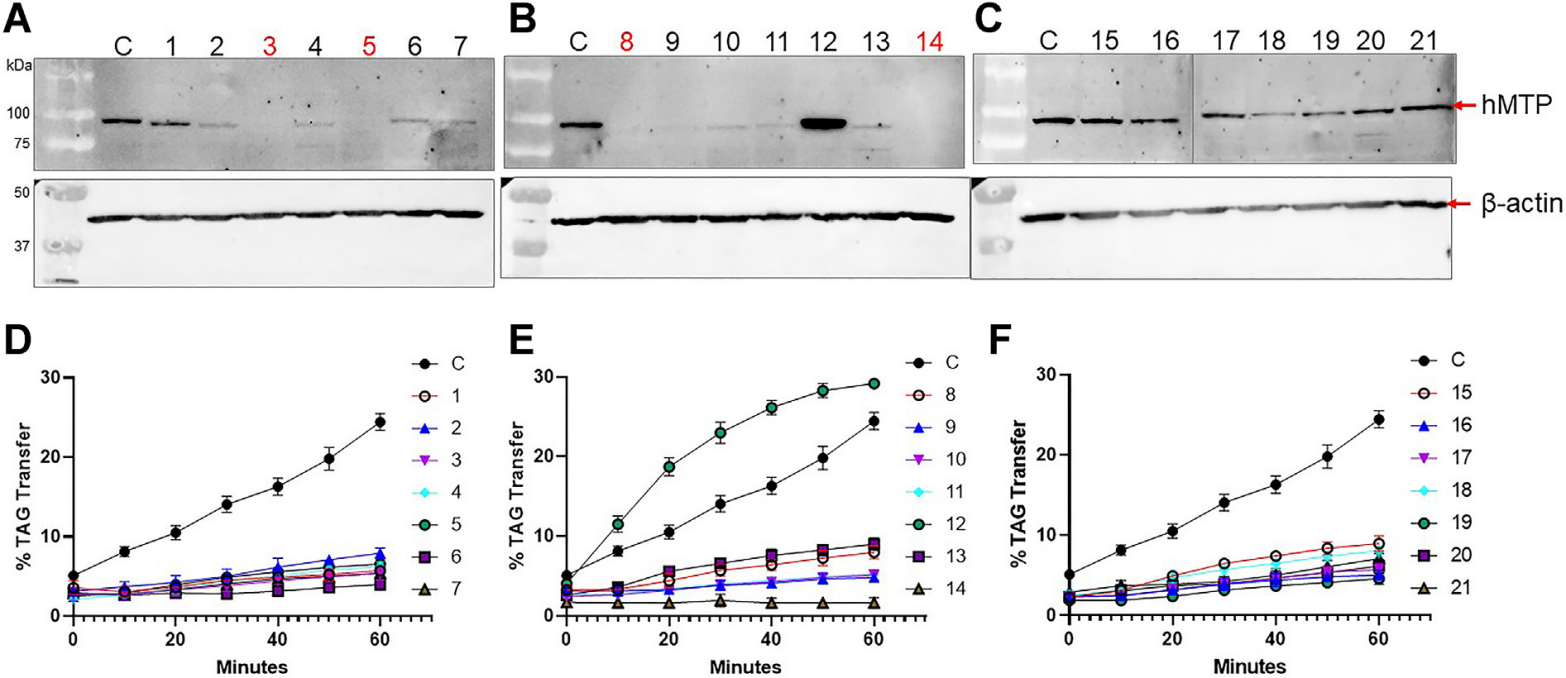
Determination of MTP protein expression and activity in selected colonies. Twenty-one colonies with no to reduced apoB secretion were transferred to 6-well plates. After reaching confluence, cells were trypsinized, and proteins were extracted after lysing the cells by sonication. The crude protein lysate (20 μg) was resolved on 8% SDS-PAGE gel and transferred to nitrocellulose membrane. The MTP was immunodetected using polyclonal anti-hMTP antibodies. (A) Colonies 1–7, (B) colonies 8–14, and (C) colonies 15–21. “C” is wild type Huh-7 cells. The colonies in which there was no detectable MTP band are in red. The TAG transfer activity of MTP in all the samples (50 μg of crude protein) was measured in triplicate. (D) Colonies 1–7, (E) colonies 8–14, (F) colonies 15–21, and “C” is Huh-7 wild type (control). apoB, apolipoprotein B; MTP, microsomal triglyceride transfer protein; TAG, triacylglycerol.

**Fig. 6:**
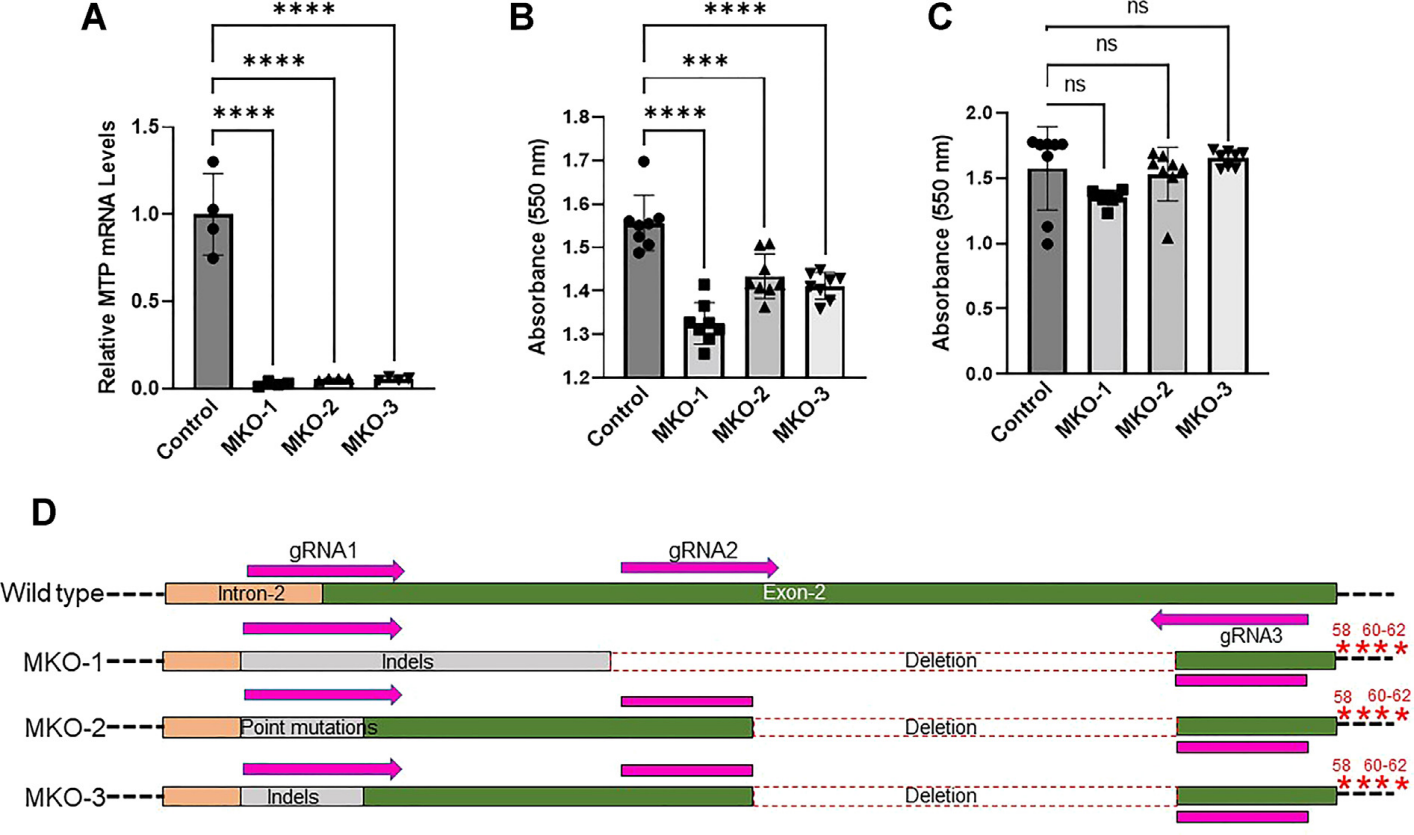
MTP mRNA levels in MKO cells, cell growth, and identification of mutations. A: The mRNA levels of MTP in clones with no detectable MTP protein were quantified by RT-qPCR. Total RNA from control and MKO cells was isolated using Trizol, and 2 μg of RNA was converted to cDNA using random primers. Appropriately diluted cDNA was used for quantitative real time PCR. *18S* was used as internal control. Relative expression of *MTP* mRNA in control (Huh-7 wild type), MKO-1, MKO-2 and MKO-3 (n = 3) cells. B, C: To determine cell growth, 10,000 cells were seeded in each well of 96-well plate and grown for 24 h and 48 h. Cells were washed and incubated with 300 μl of MTT reagent for 3 h, and purple color developed was measured at 550 nm after (B) 24 h and (C) 48 h (n = 8). The bars and error bars represent mean ± SD. To calculate the significance one way ANOVA nonparametric (multiple comparison) was used, ∗∗∗ and ∗∗∗∗ represent *P* < 0.001 and *P* < 0.0001, respectively. D: To identify mutations in these clones, exon 2 was amplified using primers 1 and 2 and sequenced. The sequence data were aligned using SnapGene to identify indels, deletions, and other mutations. Red star ∗ represents stop codon and numbers indicate amino acids in wild type MTP sequence. MTP, microsomal triglyceride transfer protein.

Although many colonies showed no or reduced apoB secretion, it was not clear whether they express MTP protein or not. Some missense mutations do not support apoB secretion despite expressing MTP protein normally (8). To understand whether these clones completely lost MTP protein expression or not, cell lysates were resolved on 8% SDS-PAGE gel, transferred to membranes, and probed with polyclonal anti-human MTP antibodies. Clones 1, 15, 16, 17, 20, and 21 showed MTP expression nearly equivalent to controls (Fig. 5A–C). Clones 2, 4, 6, 7, 9, 10, 11, and 13 showed faint bands. Clones 3, 5, 8, and 14 had no detectable MTP protein. In spite of having MTP protein expression, several clones lost TAG transfer activity (Fig. 5D–F). Clone 12 expressed higher MTP than wild type (Fig. 5B) also showed higher TAG transfer activity (Fig. 5E). The clones 3, 5, 8, and 14 did not show any detectable levels of MTP protein and very low or no TAG transfer activity (Fig. 5A, B). The clones 16–21 had equivalent expression of MTP protein compared to control but showed no TAG transfer activity (Fig. 5F). The presence of inactive protein may interfere in the quantifications of MTP protein in future studies, therefore, these clones were discarded. The MKO clones 3, 5, 8, and 14 that showed no MTP protein were carried forward. During passages MKO clone 14 did not grow well. Three clones MKO-1 (clone-3), MKO-2 (clone-5), and MKO-3 (clone-8) did grow well and were used for further characterization. The transcript levels of *MTTP* gene was reduced >90% in all three MKO clones (Fig. 6A). The reductions in MTP mRNA levels in MKO clones might be due to nonsense-mediated mRNA decay (33). These results indicate that the *MTTP* gene was knocked out in these three clones of Huh-7 cells resulting in loss of mRNA, protein, and activity.

Then, we tested the growth of these MKO cells. The growth of MKO cells was significantly slower in the first 24 h (Fig. 6B), but they caught up with the control cells within 48 h (Fig. 6C). Thus, these cells may initially show slow growth after plating, but they eventually grow well.

The above studies showed that we have successfully obtained three clones deficient in MTP mRNA, protein, and activity. Furthermore, these cells do not secrete apoB. They did not show defects in cell growth. Therefore, we used these clones for further characterization.

### Genetic basis for the loss of MTP mRNA and protein

To explain reasons for the loss of MTP mRNA and protein in these clones, we amplified exon 2 and sequenced (Fig. 6D). It appeared that sgRNA1 caused indels and point mutations as these were found in all three clones around the region recognized by sgRNA1. Indels in MKO-1 were larger in size than those in MKO-3. However, large indels were not found in MKO-2; instead, several point mutations were seen, and correct annotation of these mutations was difficult in this region. A common feature in the three clones was deletions of sequences between sgRNAs 2 and 3. This region was deleted similarly in MKO-2 and MKO-3, but MKO-1 clone had larger internal deletion. Another common feature in these clones was the introduction of stop codons at 58, 60, 61, and 62 amino acids and is possibly due to the sgRNA3. These studies suggest that sgRNA1 might have caused indels and point mutations, sgRN2 and sgRNA3 caused internal deletions, and sgRNA3 introduced early stop codons.

### Ablation of MTP increases lipid accumulation

**Fig. 7:**
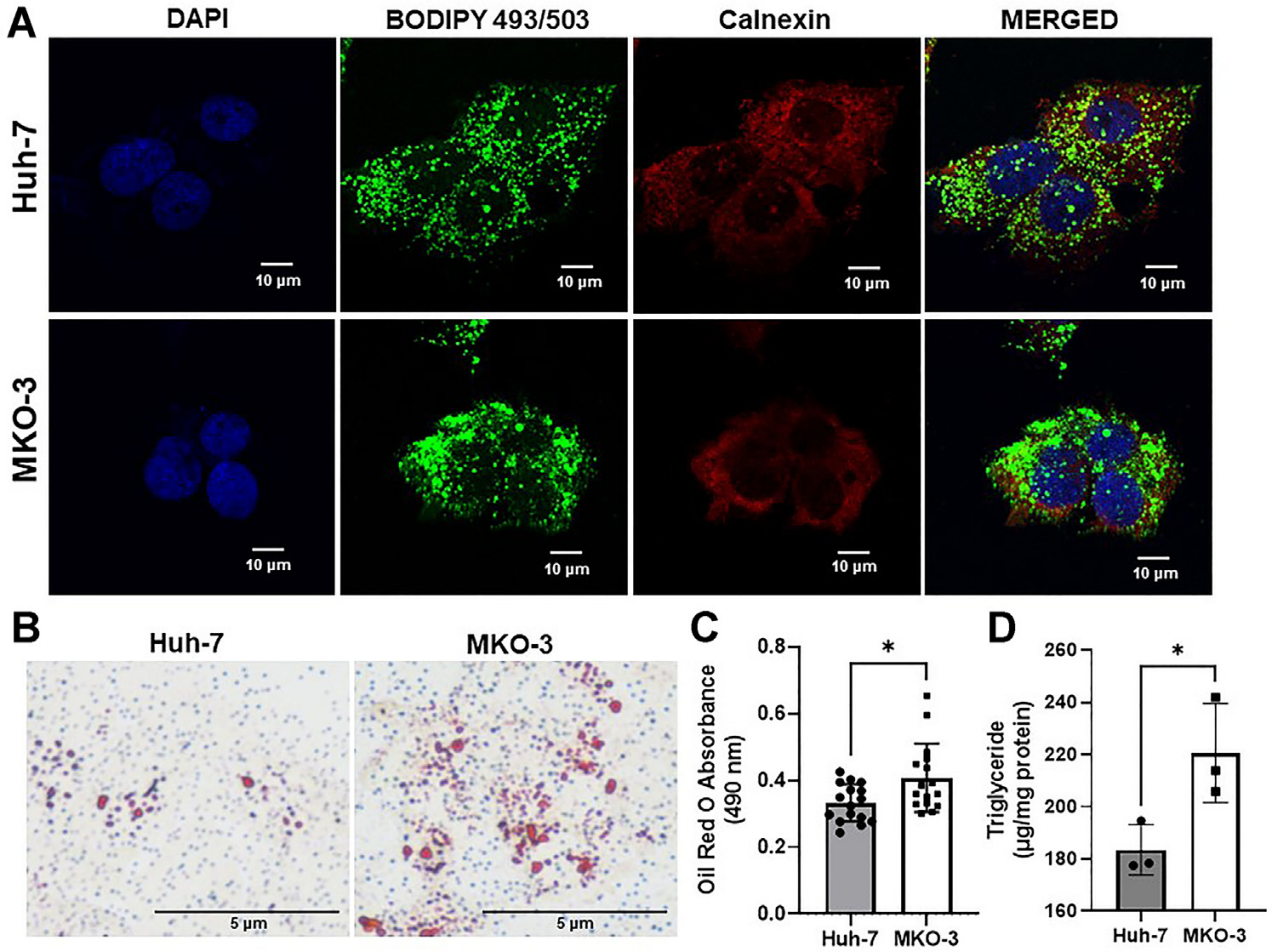
Increased lipid accumulation in MKO cells. A: Lipid droplets (green) in both Huh-7 and MKO-3 cells were stained with BODIPY 493/503 after culturing for 96 h. Endoplasmic reticulum (red) was visualized by staining for calnexin. Scale bar, 10 μm. B: Cells were stained with lipophilic Oil red O and observed under bright-field microscope. C: Oil Red O was extracted from the stained cells, and absorbance was measured. D: Total TAG was extracted using isopropanol from 96 h-old cultures and quantified colorimetrically. All data were plotted as mean ± SD and statistically analyzed by unpaired *t*-test, where ∗ represent significance at *P* < 0.05. TAG, triacylglycerol.

**Fig. 8:**
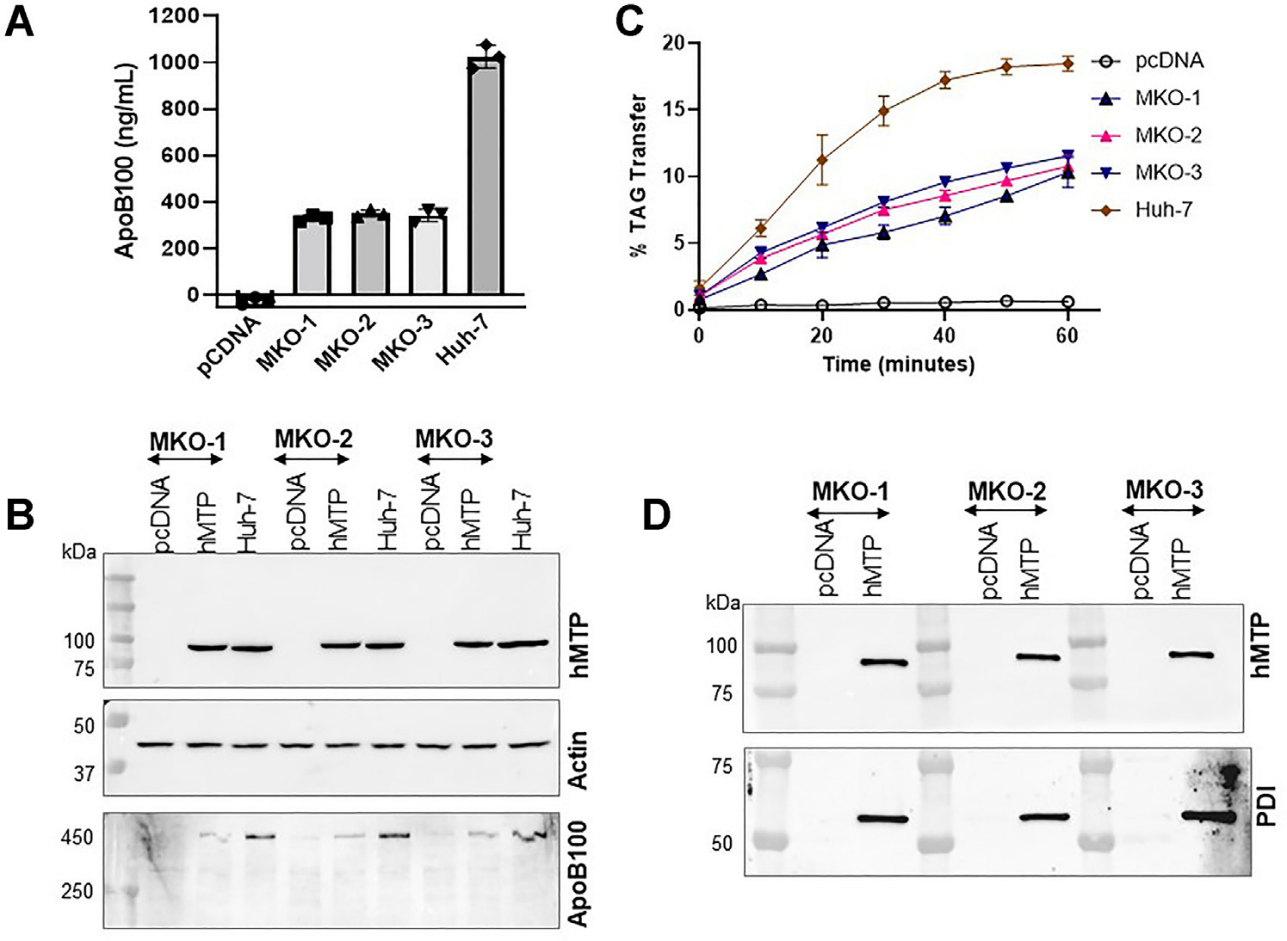
Restoration of MTP function after re-expression. Plasmids pcDNA3 or pcDNA3-hMTP-FLAG were transfected into three different MKO cell lines. After 48 h, (A) media were used to measure apoB100 secretion using ELISA. B: Cells were lysed in buffer K. Cell lysates (30 μg) were resolved on 8% SDS-PAGE and blotted on to nitrocellulose membranes. MTP protein was detected using polyclonal anti-hMTP antibodies (top). Beta-actin was used as loading control (middle). The apoB100 in media were detected by resolving 30 μl of the media on 6% SDS-PAGE gel, transferring to membrane, and probing with anti-ApoB monoclonal antibodies (1D1) (bottom). C: MTP’s TAG transfer activity was measured using 50 μg of crude cell lysate. D: The FLAG-tagged MTP protein was purified using anti-FLAG M2 affinity gel from transfected cells. The purified MTP protein was resolved on 8% SDS-PAGE gel and transferred to nitrocellulose membrane. The MTP protein (top) and its partner PDI (bottom) were detected using polyclonal anti-hMTP antibodies (Abcam) and anti-PDI monoclonal antibodies (Invitrogen), respectively. apoB100, apolipoprotein B100; PDI, protein disulfide isomerase.

It is known that MTP inhibition and ablation leads to steatosis (34, 35). Therefore, we asked whether MTP ablation and loss of apoB secretion lead to lipid accumulation. We did not see significant increases in cellular lipids after 24 and 48 h of plating. However, cells had more lipids after 96 h as determined by BODIPY staining (Fig. 7A). Moreover, when these cells were stained for the ER-specific marker calnexin, the lipid droplets did not co-localize with calnexin (Fig. 8A) indicating for the accumulation of cytosolic lipid droplets. Oil Red O staining (Fig. 7B) also showed increased lipid stain accumulation (Fig. 7C). Furthermore, these cells had more TAG (Fig. 7D) indicating that MKO cells accumulate more lipids. Therefore, these cells could also be useful in studying reversals of lipid accumulation pursuant to expression of different mutant proteins and drugs.

### Restoration of MTP activity and apoB secretion by expression of MTP in MKO cells

To understand whether we can restore the apoB secretion by expressing the MTP cDNA in MKO cells, these cells were transfected with control pcDNA3 plasmid or pcDNA3-carrying hMTP-FLAG cDNA (5, 9, 10). MKO cells transfected with pcDNA3 alone did not secrete apoB (Fig. 8A); however, transfection of all the three colonies with pcDNA3-hMTP-FLAG significantly increased apoB secretion compared with pcDNA3-transfected cells. The apoB secretion, however, was significantly lower than wild type Huh-7 cells (Fig. 8A). This is probably due to low efficiency of transfection. We have previously shown that plasmid transfection results in the expression of the transgene in 20%–30% cells (10).

Increased secretion of apoB indicated successful expression of MTP. This was further confirmed by studying expression of the MTP protein by immunodetection (Fig. 8B) or by measuring its TAG transfer activity (Fig. 8C) and association of the expressed MTP subunit with endogenous PDI (Fig. 8D). We could detect MTP protein in MKO cells transfected with plasmids expressing hMTP-FLAG similar to that present in wild type Huh-7 cells but not in cells transfected with pcDNA3 alone (Fig. 8B, top). Furthermore, MTP’s TAG transfer activity was significantly enhanced after the expression of hMTP-FLAG (Fig. 8C). The lipid transfer activity in transfected cells was about 30%–40% of that seen in wild type Huh-7 cells. To study association of MTP with the PDI subunit, we purified hMTP-FLAG using anti-FLAG M2 affinity gel chromatography (Fig. 8D). hMTP-FLAG could be purified from cells transfected with pcDNA-hMTP-FLAG but not from pcDNA transfected cells (Fig. 8D, top). The purified protein was also immunoblotted with anti-PDI antibodies (Fig. 8D, bottom). We could detect PDI in MTP protein purified from hMTP-FLAG transfected cells. These studies show that MKO cells have no detectable apoB secretion capacity, MTP protein, and triglyceride transfer activity. Overexpression of wild type MTP results in the expression of the protein that binds to endogenous PDI, transfers TAG, and supports secretion of apoB100.

## Discussion

We report successful generation of MTP-deficient human hepatoma cells using sgRNA and Cas9 RNP complex and electroporation. This method should be useful in generating Huh-7 cells deficient in other genes. Furthermore, by following a similar strategy it should be feasible to generate other cell lines deficient in specific gene(s). In this study, we selected three clones that were deficient in MTP mRNA, protein and activity, as well as in apoB secretion. These MKO cells could be useful to (1) screen MTP mutants for different lipid transfer activities and their role in apoB100 secretion in human liver cells; (2) find interacting partners of MTP protein; (3) identify drugs that can reduce liver fat accumulation; (4) discover other proteins whose synthesis and secretion may depend on MTP; and (5) investigate compensatory changes in response to loss of MTP function.

Our group studies evolution (5, 6, 7) and structure function (8, 9, 10) of MTP to identify different amino acids and structural motifs participating in the transfer of different lipids and to understand molecular bases for the missense mutations in the *MTTP* gene in abetalipoproteinemia patients. In these studies, our approach has been to transfect Cos-7, monkey kidney, cells with plasmids expressing human MTP and apoB48 to study the role of MTP mutations in apoB secretion. A disadvantage of this cell culture system is that both MTP and apoB plasmids need to be transfected. Another disadvantage is that this system is not amenable to study the role of MTP in apoB100 secretion due to difficulties in expressing large apoB100 polypeptide in these cells (12). Therefore, we hypothesized that knockout of the endogenous *MTTP* gene in hepatoma cells may avoid the need to transfect cells with large apoB100 expressing plasmids for secretion studies. Further, these liver-derived cells may provide a better platform to address the role of different human MTP mutations in the secretion of apoB100-containing lipoproteins than kidney cell lines. Therefore, we used two different methods to establish MTP-deficient human hepatoma cell lines.

First, we tried to generate knockout cell lines using plasmid-based expression of gRNA and CRISPR/Cas9 with cotransfection of HDR plasmid containing flanking sequences of Cas9 cutting sites, puromycin resistance, and *rfp* genes. Although we were successful in obtaining puromycin resistant, RFP-positive colonies, these cells expressed MTP. Thus, this approach was not successful in ablating *MTTP* gene from Huh-7 cells. This was not due to improper selection of gRNAs as the same gRNAs gave MTP-deficient cells using electroporation (see below). A reason for the failure in obtaining MTP deficient cells may be low transfection efficiency of plasmids that usually ranges between 20% and 30% (8, 9, 10, 12). Another reason for the failure might be the heterogeneous nature of Huh-7 cells that carry an average of 60 chromosomes (36). Therefore, we reasoned that it might be difficult to generate a complete knockout of all *MTTP* alleles using plasmid-based method and sought alternate methods for gene knockout. To this end, we used in vitro-formed RNP complexes containing synthetic sgRNAs that target MTP exon 2 and active Cas9 protein. This RNP complex was delivered to Huh-7 cells by electroporation using different electromotive forces. The efficiency of MTP knockout was assessed by measuring apoB secretion. The highest efficiency was achieved with 1,650 V pulse. After 1,650 V pulse, a majority of cells lost apoB secretion capacity, whereas cells transfected using 1,450 V and 1,250 V reduced apoB secretion by 70% and 15%, respectively. The PCR amplification of the targeted MTP exon 2 sequence from RNP transfected cells using 1,650 V and 1,450 V showed diffused bands indicating disruption of the *MTTP* gene. Subsequently, we isolated single cell colonies for further studies.

We selected three clones for further studies that had no detectable MTP mRNA, protein, and activity, so that residual missense protein does not interfere in future studies. The *MTTP* gene ablation in these clones was confirmed by several ways. The selected MKO cells did not have measurable MTP mRNA, protein, and activity. They were deficient in supporting apoB secretion. These cells accumulated more lipids with time. We defined indels, internal deletions, and stop codons explaining reasons for the absence of MTP mRNA and protein. More importantly, these defects appear to be solely due to MTP deficiency as re-expression of MTP via plasmid transfection resulted in the binding of the expressed MTP with the endogenous PDI subunit, partial restoration of TAG transfer activity and apoB secretion.

Further characterization of these MKO cells showed slower growth in the initial 24 h; however, by 48 h, their growth was similar to wild type Huh-7 cells. These results suggest that MTP knockout does not have severe deleterious effect on cell growth. Second, we studied the accumulation of lipids in these cells. It is known that MTP deficiency leads to accumulation of lipids in cells. We found that MKO cells accumulated more lipids than control cells after 72 h. Thus, these cell lines grow well and accumulate more lipids with culture time.

In conclusion, the synthetic RNP complex in combination with electroporation is more efficient tool to make gene knockouts in cell lines. This method can be used to generate specific gene-deficient cell lines. Using this method, we effectively ablated the human *MTTP* gene in Huh-7 cells and successfully propagated individual MKO clones that express no MTP mRNA, protein, and activity as well as show no apoB secretion. These MTP-deficient stable cell lines may be useful in studying mutations in intron-exon junctions and other major changes after the expression of mutants. Besides being very useful to study protein stability and lipid transfer defects, these cells may be helpful in identifying partners that may interact with MTP and other proteins that may depend on MTP for their synthesis and secretion. Furthermore, these cells may be useful in identifying compensatory changes secondary to loss of MTP function.

## Data availability

All the data are included in the manuscript.

## Conflict of interest

The authors declare that they have no conflicts of interest with the contents of this article.
